# Critically assessing the idea of wildfire managed retreat

**DOI:** 10.1088/1748-9326/ad31d9

**Published:** 2024-03-28

**Authors:** Kathryn McConnell, Liz Koslov

**Affiliations:** 1 Brown University, Population Studies and Training Center, Providence, RI 02912, United States of America; 2 University of California, Los Angeles, Department of Urban Planning and Institute of the Environment and Sustainability, Los Angeles, CA 90024, United States of America

**Keywords:** managed retreat, wildfire, planned relocation, climate change adaptation

The rapid growth of wildfire destruction around the world [1] and model projections of intensifying fire impacts under climate change [2] raise critical questions over the habitability of fire-prone regions. While wildfires have always been and remain a vital part of natural ecosystem functioning [3], the rising impacts of rare but highly destructive wildfires pose new challenges. In response to changing wildfire regimes, calls for managed retreat—the intentional relocation of built infrastructure away from hazardous areas [4]—have emerged in public discourse around wildfires.

Managed retreat initiatives are well-documented as a response to flooding, and include community-initiated relocation of infrastructure and household-level property buyouts [4–6]. By contrast, projects that could be considered ‘wildfire retreat’ are far rarer in practice and have received limited analytical attention. Academic researchers have suggested that retreat can be an adaptive response to wildfire [7–9], but few have investigated whether it is effective, equitable, or politically feasible in this hazard context. Absent rigorous empirical study and evaluation, simply applying a flood-based approach to a wildfire context could prove maladaptive.

We are at a juncture where substantial funding is being directed toward the wildfire crisis. In the United States, recent federal legislation designated billions of dollars for wildland fire mitigation, and wildfire experts have called for further investments into the built environment and residential communities [10]. The growth of annual wildfire destruction coupled with major government spending to address this threat make the stakes of how we conceptualize wildfire retreat extremely high.

We lay out a research agenda to critically evaluate managed retreat as an adaptive response to wildfire. While there are lessons to be drawn from flood-based managed retreat, important differences exist between flooding and wildfire contexts, underscoring the need for a distinct research agenda. The proposed line of analysis will build a more geographically expansive understanding of climate relocation planning and will clarify the potential for retreat to be used as a form of wildfire adaptation. This research is needed to ensure that managed retreat approaches are not bluntly applied across hazard contexts without attending to the specificities of fire-prone places and the communities that reside there.

## 1. Wildfire managed retreat scenarios

This novel research agenda focuses on three primary wildfire retreat scenarios that are in line with the widely accepted understanding of managed retreat as involving the withdrawal of built infrastructure from areas at high hazard risk and subsequent repurposing of land [4]. First, individual residents and entire communities might seek to preemptively relocate away from fire-prone places, leaving vacated land as non-residential. Second, governments might seek to acquire and repurpose land or limit reconstruction in places where a wildfire has destroyed buildings. Third, infrastructural wildfire retreat could seek to remove or relocate the infrastructures and land use types that heighten wildfire risk, such as above-ground power lines and certain forms of plantation forestry. These modes of wildfire retreat each carry their own constellation of social, political, and governance considerations, requiring nuanced examination.

We distinguish these wildfire retreat scenarios from related but distinct dynamics of wildfire and the built environment: avoidance, institutional withdrawal, and displacement. Zoning and land use regulations have been used to prevent new building construction in undeveloped high fire risk areas [10]. Such approaches, referred to as ‘avoidance’ [11], do not remove infrastructure or housing, but rather maintain the existing state of undeveloped lands. Meanwhile, the cessation of homeowners’ insurance in hazardous regions or government disinvestment from critical infrastructure such as road networks can lead to de facto or ‘unmanaged retreat.’ While these forms of institutional withdrawal may eventually result in the abandonment of residential lands, neither involve the deliberate investment *into* infrastructure removal, land repurposing, and/or supported relocation that define managed retreat. In keeping with the work of flood-based retreat scholars [12], we do not consider situations in which wildfire destroys residents’ homes—causing people to permanently relocate with minimal planning or support—to be a form of managed retreat. This is displacement and, like avoidance and withdrawal, should be considered analytically distinct.

While we propose a focused working definition of wildfire managed retreat to guide research, we recognize that retreat is likely to occur in conjunction with other changes in the built environment, including avoidance and institutional withdrawal as well as wildfire mitigation efforts that support adaptation in place. Moreover, retreat initiatives such as government property acquisitions have often been spurred by prior displacement events, complicating a clear distinction between the two. For example, one of very few documented government buyout initiatives to target wildfire-prone properties was only established in response to Australia’s highly destructive 2009 Black Saturday Bushfires [13]. We acknowledge this broader context, while focusing our research agenda on forms of retreat that involve the investment of resources and an intentional transformation of land use.

## 2. Primary research needs: communities, land, spatial dynamics, & cultural politics

First, research is needed to examine how the specific social and political dynamics of communities exposed to wildfire shape the potential for managed retreat. Existing research points to important geographic differences between fire-prone places and the locations where flood retreat initiatives have historically been concentrated. In the United States, the greatest wildfire potential tends to occur in suburban, exurban, and rural regions [14], while most flood-based property buyouts have occurred in more urbanized settings [5]. In addition to these differing geographies, there are also likely to be differences between how fire- and flood-affected populations respond to retreat initiatives. While scholars have described community-initiated retreat from floods [4, 6], there is scarce comparable research demonstrating collective efforts to relocate in a wildfire context. In fact, existing research on wildfire-related mobility emphasizes residents’ desire to remain in place [15].

Future research should document the long-term visions of residents living in fire-affected places to build a deeper understanding of their distinct mobility aspirations and capabilities. Beyond investigating risk perceptions, there is a need for research that examines whether different residents seek to remain in place or to relocate from fire-prone areas, and whether they have the resources to realize these aims. Particular consideration is needed of the plans and aims of Indigenous communities who reside and/or have homelands in fire-prone places. Researchers should further investigate the political dynamics of managed retreat initiatives in different settings, and how coalitions converge around remaining in place or retreating. Whether managed retreat proposals hold broad political support in a given community is a separate, but equally important, question from more technical policy considerations.

Second, research must seriously consider the land management implications of wildfire retreat efforts. Flood-based retreat initiatives generally do not plan or budget for land management in the years and decades *after* retreat. Instead, post-retreat land often transitions from residential occupation to being ‘unmanaged’ [9]. But past land use histories suggest that the movement of people away from fire-prone places does not necessarily lead to reduced fire risk. For instance, in Mediterranean regions of Europe, the depopulation of rural, agricultural regions during the mid-twentieth century and subsequent land abandonment resulted in more severe wildfires due to fuel buildup [16]. Researchers should evaluate the potential impacts of more unmanaged land on local fire risk, especially if it is interspersed within or immediately adjacent to residential areas. Depending on how it is managed, unoccupied post-retreat land could potentially heighten rather than reduce fire risk.

Research should further explore what existing land stewardship practices and emerging nature-based initiatives may be well-suited to wildfire retreat. Given the importance of Indigenous cultural burning to fire management and maintaining ecosystem health [10], what role might Indigenous stewardship play for post-retreat land? Another area of growing interest is the strategic repurposing of land into protective buffers. For example, following the 2018 Camp Fire, the Town of Paradise, California began acquiring formerly residential properties and pooling them to establish a collective buffer [9]. In China, low-flammability plant species have been strategically planted as “green firebreaks” [17]. These practices could be evaluated as approaches to stewarding post-retreat land.

Third, researchers and practitioners need a clearer picture of how the spatial dynamics of wildfire shape the feasibility of managed retreat. Embedded in flood-based retreat efforts is the assumption that certain spatial zones of risk can be delineated and projected into the future with relatively high confidence. Managed retreat policies then remove built infrastructure from these risk zones. The highly dynamic nature of fire probability complicates this approach for wildfire retreat. As a landscape changes, so too does wildfire risk. Land management practices, vegetation patterns, and the built environment all strongly influence wildfire dynamics [18, 19]. Thus, spatial zones of wildfire risk can and do shift over time, posing a moving target for retreat efforts. Furthermore, the wildland-urban interface (WUI)—the land use type that generally poses highest fire risk to residents—is widespread and diffuse, encompassing vast landscapes and large numbers of structures. As of 2020 in the continental United States, an estimated 44.1 million houses were located in this land cover class [18]. While managed retreat may be a plausible strategy for targeted interventions, the enormous spatial extent of wildfire exposure poses fundamental scalar problems for adopting retreat as a primary policy response.

Fourth, and relatedly, the proposition of wildfire retreat invites the question of whether, rather than relocating residential structures or people, policymakers might instead consider retrofitting or relocating risk-producing infrastructures and land uses. Power lines, for instance, have ignited a substantial number of high-severity wildfires in the United States [20] and Australia [21]. In fires documented across countries such as Portugal, Chile [22], and South Africa [23], destruction to housing has been facilitated by rapid fire spread through adjacent commercial forestry plantations. Efforts to curtail flammable plantation forests or to underground power lines offer a different approach to retreat, one that targets infrastructures and land uses that produce risk rather than the residents who are exposed to it. Such infrastructural retreat would involve deliberate investment into infrastructure reconfiguration, by contrast to the institutional withdrawal observed in disinvestment from plantation management and power line maintenance.

The idea of infrastructural retreat highlights broader spatial questions around the production of hazard risk. Rather than focusing exclusively on the geographies where hazard exposure occurs, retreat scholars should explore how wildfire risk is generated through connections to spatially distant places. Plantation forestry, for instance, is embedded in larger commodity chains and power lines can be connected to regional energy networks, both of which link commodity producing and consuming regions [24]. Emerging wildfire research is taking such land teleconnections seriously, for instance by documenting the relationship between development dynamics in urban cores and housing expansion in spatially distant, periurban and rural regions [25]. Future research should consider how the ties between places—constituted through trade, energy, and migration flows—unevenly drive wildfire risk across the urban-rural gradient, with implications for the effectiveness of managed retreat.

Finally, research is needed to analyze the cultural politics of emerging wildfire managed retreat discourses, even in the absence of these projects proving feasible or desirable on the ground. How do scientific and public narratives frame the causes of growing wildfire destruction, and therefore what should be done about it? Is wildfire destruction blamed on government land management practices? On major carbon emitters? On rising housing costs in urban cores? Or, are residents of fire-prone places portrayed as simply having made ‘bad climate decisions’ [26]? This line of research should critically attend to the ways that certain wildfire retreat proposals build on narratives of inevitable rural decline and urban ascendance [27]. The discourses that develop around wildfire attribution and affected residents’ deservingness of government support carry important implications for climate justice. They will shape policy priorities, including whether and how managed retreat is advanced as a possible solution.

## 3. Conclusion

Scientific research on the prospect of managed retreat from wildfires is needed, but cannot alone answer the fundamentally normative questions posed by a changing climate. How *should* resources be allocated to people living and working in the places most affected by climate-related hazards? Who bears responsibility for these costs? Rather than treating managed retreat exclusively as a technical scientific exercise, the line of scholarship we propose should consider the histories of land governance and urbanization that produced current patterns of wildfire exposure and vulnerability, and explicitly engage with the ethical dilemmas posed by amplifying wildfire impacts.

Managed retreat is not necessarily an appropriate response to fire risk, nor is it the only alternative to wildfire-induced displacement. We argue that managed retreat strategies should be examined as one possible approach alongside a range of *in situ* adaptations that allow residents to more safely live with fire, and alongside policy changes that target the drivers of growing fire risk. Failing to address interconnected questions of housing, land use, and wildfire leaves communities and governments in an increasingly dangerous and costly status quo: continued waves of structure loss, displacement, reduced housing stock, and enormous expenditures for the public and private sectors. In collaboration with diverse fire-affected communities, managed retreat initiatives should be critically evaluated as a response to wildfire risk. Such research will help to avoid maladaptation, and offers opportunities to generate novel, community- and nature-based solutions to the wildfire crisis.

## Data Availability

No new data were created or analysed in this study.
